# Genetic diversity and polymorphism of genes associated with meat quality and growth traits of A Luoi Yellow cattle in Vietnam

**DOI:** 10.14202/vetworld.2024.2295-2303

**Published:** 2024-10-17

**Authors:** Thu Nu Anh Le, Huong Thi Duong, Phuong Thi Lan Le, Thuong Thi Nguyen, Thuan Thi Duy Phan, Phung Dinh Le, Van Huu Nguyen

**Affiliations:** 1 Department of Animal Science, Faculty of Animal Science and Veterinary Medicine, University of Agriculture and Forestry, Hue University, Hue City, Vietnam; 2 Department of Clinical Veterinary Science, Faculty of Animal Science and Veterinary Medicine, Nong Lam University, Ho Chi Minh City, Vietnam

**Keywords:** A Luoi yellow cattle, growth trait, indels, meat tenderness, single nucleotide polymorphisms

## Abstract

**Background and Aim:**

A Luoi Yellow cattle is an indigenous cattle breed that is raised in the A Luoi District, Vietnam, characterized by its small body size, high adaptability, and meat quality favored by domestic consumers. Marker-assisted selection is an effective approach for improving breeding genetics and sustainably developing livestock production. Therefore, this study aimed to investigate the genetic diversity and polymorphism of genes associated with meat quality and productivity in the A Luoi Yellow cattle population with the goal of future breeding selection and sustainable development of the A Luoi Yellow beef brand.

**Materials and Methods:**

In this study, we genotyped six functional genes, including Leptin (*LEP*), Calpastatin (*CAST*), Calpain 1, pleomorphic adenoma gene 1, Sirtuin 1, and Sirtuin 2 (*SIRT2*), involved in meat quality and growth traits using polymerase chain reaction (PCR) or PCR-restriction fragment length polymorphism. We also investigated mitochondrial DNA (mtDNA) and the Y chromosome-specific gene on the Y chromosome to elucidate the genetic diversity and paternal and maternal origin of the A Luoi Yellow cattle using Sanger sequencing.

**Results:**

The results showed that A Luoi yellow cattle have *Bos indicus* origin from both paternal and maternal lineages. By mtDNA analysis, we identified two new haplotypes of the I1 haplogroup that were not previously detected. The genotyping of the six functional genes indicated that A Luoi Yellow cattle carry favorable alleles that increase meat tenderness and body size, with frequencies of 0.02–0.40. In particular, the presence of desirable homozygous genotypes of the *CAST*, *LEP*, and *SIRT2* genes will be important for the future selection of animals based on their potential performance in meat quality and productivity.

**Conclusion:**

The findings of this study is useful for the future breeding and sustainable development of A Luoi Yellow cattle.

## Introduction

Yellow cattle are a small native breed in Vietnam with inferior productivity that cannot take advantage of economies of scale in breeding and marketing programs, thus putting them at risk of gradual reduction in genetic variants [1]. However, due to their high adaptation to local tropical environmental conditions, Yellow cattle are raised on a small scale in poor rural regions in Vietnam. A Luoi is a high mountainous district of Thua Thien Hue Province, Vietnam, which has a diversity of natural resources with 49% of the area being protected forests, 47% being agricultural land, including forest for production and grassland for livestock raising [2]. Owing to that diversity, most local people can earn their living by engaging in agricultural activities. In particular, raising domestic animals improves the livelihoods of rural households by taking advantage of available food sources. Yellow cattle are popularly raised in A Luoi by natural grazing, and beef is favored by domestic consumers. Because of these advantages, the A Luoi local government and farmers have attempted to build an A Luoi Yellow beef brand, and last year, the trademark “A Luoi Yellow Beef” was granted a national protection certificate by the Intellectual Property Department of the Ministry of Science and Technology [3]. Therefore, improving the meat quality and performance of A Luoi Yellow cattle is necessary for the sustainable development of this breed and the livelihoods of A Luoi farmers. In addition, determining the genetic diversity in the Yellow cattle population in A Luoi is also crucial for answering the question of whether there is an introgression of the taurine genome in this population since taurine cattle are well known for their high production and tenderness. However, since the molecular genetic characteristics of A Luoi Yellow cattle, including origin, meat quality, and growth traits, have not been clarified, it is essential to access advanced molecular genetic technologies for breeding selection programs of Yellow cattle in the A Luoi District, Vietnam.

Convincing evidence has shown that genotyping technologies in animal breeding programs can detect the genetic diversity and desirable alleles of the desired traits; hence, they are commonly used for breed conservation and increase the accuracy of breeding selection [4–6]. In addition, the combination of traditional and genomic information in breeding programs has been proven to lead to improvements in small- to medium-scale breeds [7]. For example, using genotyping technologies and phenotype records, novel single-nucleotide polymorphisms (SNPs) in Leptin (*LEP*), Calpain1 (*CAPN1*), Calpastatin (*CAST*), pleomorphic adenoma gene 1 (*PLAG1*), Sirtuin 1 (*SIRT1*), and Sirtuin 2 (*SIRT2*) genes have been detected to be associated with meat quality and growth traits [8–13] in domestic animals. These SNPs have been suggested as molecular markers for breeding selection. Moreover, the D-loop region of mitochondrial DNA (mtDNA) and the Y chromosome-specific gene (*SRY*) are powerful tools for studying origins and genetic diversity. The frequency and geographical distribution of mtDNA and Y-chromosome haplotypes reveal population relationships and sex-specific gene flow and migration [14–17].

From this perspective, we investigated the genetic diversity and polymorphism of genes associated with meat quality and growth traits of A Luoi Yellow cattle in Vietnam, aiming to explore the molecular genetic characteristics of A Luoi Yellow for the future breeding selection of this cattle as well as the sustainable development of the A Luoi Yellow beef brand.

## Materials and Methods

### Ethical approval

Ethical approval from University of Agriculture and Forestry, Hue University to conduct this study was not required. However, the sample collection was performed as per the standard sample collection procedure without any harm to animals**.**

### Study period and location

This study was conducted from January 2023 to April 2024 at the Faculty of Animal Science and Veterinary Medicine, University of Agriculture and Forestry, Hue University, Vietnam.

### Animals and DNA extraction

In total, 46 blood samples of yellow cattle, including 12 males and 34 females, were collected from small livestock households in A Luoi District, Thua Thien Hue Province, Vietnam. Blood samples (3 mL) were collected from the jugular vein using heparin vacuum tubes. DNA was extracted from whole blood cells using the standard phenol-chloroform method. The concentration of extracted DNA was measured using a Nanodrop One (ThermoFisher Scientific, US).

### Primer design and polymerase chain reaction (PCR) amplification of genes associated with meat quality and growth traits

For PCR amplification, primer pairs were designed based on the bovine genomic sequences in GenBank, as listed in Table-1 [18–20], using the Primer Blast tool in National Center for Biotechnology Information (NCBI) [21] and were synthesized by Integrated DNA Technologies, Inc., USA.

**Table-1:** Primer sequences, fragment lengths, and annealing temperatures for PCR amplification.

Gene	Primer sequences (5’–3’)	Length (bp)*	Temp**	Reference seq***
*LEP*	F: GTGGATGCGGGTGGTAATG	284	57	NC_037331.1
	R: CACGGTTCTACCTCGTCTCC			
*CAST*	F: TTGGAAAACGATGCCTCACG	180	57	NC_037334.1
	R: GTCTGTCCAGGGTCTCTACGA			
*CAPN1*	F: ACT TGC TGG AGA GGG AAG GT	777	58	NC_037356.1
	R: TTC GAG CCC AAC AAG GAA GG			
*PLAG1*	F: ACTTTCCCCTTGAACCACCA	337	57	NC_037356.1
	R: CTTGTTCCGCCTCCACTTCAG			
*SIRT1*	F: AGT GGG TGT GGA GGT AGG T	419	59	NC_037355.1
	R: GTC TTC AAA TCA GTG CCC GC			
*SIRT2*	F: CCCAACCTACTTGCCGATGA	176	57	NC_037345.1
	R: GCAGGTGGCTTCCCCAAATA			
*mtDNA*	F: CTGCAGTCTCACCATCAACC	650	58	[18, 19]
	R: CCTTTGACGGCCATAGCTGA			
*SRY*	F: CCGGGCTATAAATATCGACCTC	1,062	58	[20]
	R: GATGAAACCTTGGGTCTCACAG			

* Lengths of amplified fragments.

** Annealing temperature.

*** Reference sequence for the primer design of the ARS-UCD2.0 *Bos taurus* genome. PCR=Polymerase chain reaction, SNPs=Single nucleotide polymorphisms, *LEP*=Leptin, *CAPN1*=Calpain1, *CAST*=Calpastatin, *PLAG1*=Pleomorphic adenoma gene 1, *SIRT1*=Sirtuin 1, *SIRT2*=Sirtuin 2

These primer pairs amplify the portion of candidate genes, namely *LEP* R25C, *CAST* g.2959 A>G, *CAPN1 g*. 4684 C>T, *PLAG1* Del_19bp, *SIRT1 g*. −382 G>A, and *SIRT2 g*. 17578 A>G.

PCR reactions were carried out in 10 μL reaction mixtures containing 10 ng of genomic DNA, 0.2 μM primers, 0.2 mM dNTP, 2.5 mM MgCl_2_, 5 × PCR buffer, and 1.25 U GoTaq Hot Start polymerase (Promega, USA) for 35 cycles of denaturation at 94°C for 30 s, annealing at the temperatures indicated in Table-1 for 30 s, and extension at 72°C for 60 s.

### Genotyping of candidate genes using PCR or restriction fragment length polymorphism (RFLP)

For genotyping, the digestion of PCR products was conducted using PCR-RFLP technique to detect polymorphisms of SNPs *LEP* R25C, *CAST* g. 2959 A>G, *CAPN1 g*. 4684 C>T, *SIRT1 g*. −382 G>A, *SIRT2 g*. 17578 A>G. The NEB cutter 3.0 tool (New England Biolabs, USA) [22, 23] was used to determine the restriction enzymes for the digestion of PCR products. The PCR products were incubated with restriction enzymes, as described in Table-2, following the instructions of the manufacturer (New England Biolabs, USA).

**Table-2:** Restriction enzyme and fragment lengths for PCR-RFLP genotyping.

Gene	Restriction enzyme	Fragment length (bp)**		

		NN	NM	MM
*LEP*	*Aci*I	174, 103, 7	174, 103, 7, 277	7, 277
*CAST*	*Dde*I	61, 119	61, 119, 180	180
*CAPN1*	*Bts*CI	130, 178, 470	130, 178, 470, 600	178, 600
*SIRT1*	*Ssp*I	419	419, 293, 126	293, 126
*SIRT2*	*Hpy*188III	176	176, 100, 76	100, 76

** Fragment lengths cut by restriction enzyme. NN=Normal genotype, NM=Heterozygous genotype, MM=Alternative homozygous genotype, PCR=Polymerase chain reaction, SNPs=Single nucleotide polymorphisms, *LEP*=Leptin, *CAPN1*=Calpain1, *CAST*=Calpastatin, *PLAG1*=Pleomorphic adenoma gene 1, *SIRT1*=Sirtuin 1, *SIRT2*=Sirtuin 2

Genotyping of *PLAG1* was conducted by directly observing the bands of the PCR products. After PCR amplification and PCR-RFLP, the PCR and restriction fragments were electrophoresed in agarose gels containing 2% and 3% TAE buffer, stained with Ultra Gelred (Vazyme, China), and visualized using an ultraviolet transilluminator (Bio-rad Gel Doc XR^+^, USA).

Genotype distribution and allele frequencies were calculated. Chi-square values were assessed to verify the Hardy-Weinberg equilibrium (HWE) status.

### Analysis of the parental origin of A Luoi Yellow cattle using mtDNA and the *SRY* gene

To determine the mtDNA and *SRY* haplotypes, a 650-bp fragment of the D-loop region of mtDNA and a 1062-bp segment of the *SRY* gene in the male-specific region of the cattle Y chromosome were amplified using previously described primer pairs [18–20] (Table-1). The amplified fragments were directly sequenced using these primers. PCR reactions were conducted as described above. The amplified products will be sequenced using the Sanger sequencing method.

The obtained sequences were aligned using theClustal W tool and then constructed into a phylogenetic tree using the neighbor-joining method in MEGA11 software (https://www.megasoftware.net/) [24]. These sequences were deposited in GenBank NCBI with accession numbers (PP857296–PP857341).

## Results

### Polymorphisms of genes associated with meat quality and growth traits

In the present study, we investigated the polymorphism of genes associated with meat quality and growth traits, including *LEP*, *CAST*, *CAPN1*, *PLAG1*, *SIRT1*, and *SIRT2*, in A Luoi yellow cattle using PCR or PCR-RFLP. As the results of genotyping, two genotypes were observed in *CAPN1*, *PLAG1*, and *SIRT1* genes, whereas three genotypes were observed in *LEP*, *CAST*, and *SIRT2* genes, indicating that both alleles of these genes are presented in the A Luoi Yellow cattle (Table-3 and Supplemental Figures). In addition, the population carrying these SNPs was in HWE because of lower χ^2^ values than χ^2^ (0.05; 1) at 3.84. Among six SNPs and indels detected in the A Luoi Yellow cattle, we found that most wild-type alleles had higher allelic frequencies in the population than the mutation alleles, except for SNP g. 2959 A>G in *the CAST* gene. The frequency of the alternative allele was higher than that of the wild allele.

**Table-3:** Genotype distribution and allele frequencies of SNPs associated with meat quality and growth traits.

Traits	Gene	SNPs/Indels	Genotype distribution			Allele frequencies		Chi-square values for the HWE test
Tenderness	*CAST*	c. 2959 A>G	AA	AG	GG	A	G	2.5
			10	24	12	0.4	0.6	
	*CAPN1*	c. 4684 C>T	CC	CT	TT	C	T	1.75
			31	15	0	0.84	0.16	
Growth traits	*PLAG1*	Del_19 bp	WW	WD	DD	W	D	0.02
			44	2	0	0.98	0.02	
	*SIRT1*	-382 G>A	GG	GA	AA	G	A	0.31
			39	7	0	0.92	0.08	
	*SIRT2*	g. 17578 A>G	TT	TC	CC	T	C	0.08
			35	10	1	0.87	0.13	
Meat quality and growth traits	*LEP*	R25C	CC	CT	TT	C	T	0.87
			21	18	7	0.65	0.35	

HWE=Hardy-Weinberg equilibrium, SNPs=Single nucleotide polymorphisms, *LEP*=Leptin, *CAPN1*=Calpain1, *CAST*=Calpastatin, *PLAG1*=Pleomorphic adenoma gene 1, *SIRT1*=Sirtuin 1, *SIRT1*=Sirtuin 2

Regarding SNPs associated with meat quality, including *CAST* g. 2959 A>G and *CAPN1 g*. 4684 C>T, the desirable allele frequencies of these SNPs were 0.40 and 0.16, respectively. These frequencies are lower in the A Luoi Yellow breed than in other taurine and indicine cattle breeds. Higher frequencies of the favorable allele A of *CAST* g.2959 A>G were found in Angus (0.652), Hereford (0.722), Chinese Qinchuan (0.75), and Chinese Jinnan cattle (0.677) [25]. Sun *et al*. [26] reported higher T allele frequencies of *CAPN1 g*. 4684 C>T in Simmental, Leiqiong, and Yunnan Yellow cattle, 0.484, 0.40, and 0.192, respectively.

In terms of SNPs involved in growth traits, including *PLAG1* Del_19bp, *SIRT1 g*. -382 G>A, and *SIRT2 g*. 17578 A>G, the desirable allelic frequencies of these SNPs were 0.02, 0.08, and 0.13, respectively. The frequency of the deletion (D) allele of *PLAG1* Del_19bp is distributed over various 0.0–0.95 in 37 cattle breeds raised at different latitudes [27]. With the increase in the latitude of the cattle breeds, the wild (W) allele frequency also had an increasing trend. In which, Tibetan cattle that were raised in the area of high altitude and low latitude (29°) did not have D allele in the population. The A Luoi is a high mountainous district of Vietnam located at a latitude of 16°, and the D allele frequency of the A Luoi Yellow cattle is quite low, as reported in these reports. A higher frequency of the desirable A allele of *SIRT1 g*. −382 G>A was observed in Luxi cattle at 0.278 [28]. The frequencies of the favorable allele G of *SIRT2 g*. 17578 A>G were also low in other indicine native cattle breeds, ranging from 0.086 to 0.142 [29].

Moreover, previous research by Almeida *et al*. [30], and Kononoff *et al*. [31] have reported that the polymorphism of *LEP* R25C is associated with both meat quality and growth traits. In this study, the frequency of favorable T alleles was 0.35 in the A Luoi Yellow cattle. Higher T allele frequencies of *LEP* R25C in taurine-type breeds, such as Angus, Hereford, Gelbvieh, Limousine, and Charolais are observed with 0.71, 0.55, 0.48, 0.77, 0.42, and 0.52, respectively [32]. However, this frequency in A Luoi Yellow cattle was higher than that in another zebu breed (0.115 of T allele in Kebumen Ongole Grade cattle) [33].

### The parental origins of the A Luoi Yellow cattle

**Figure-1:**
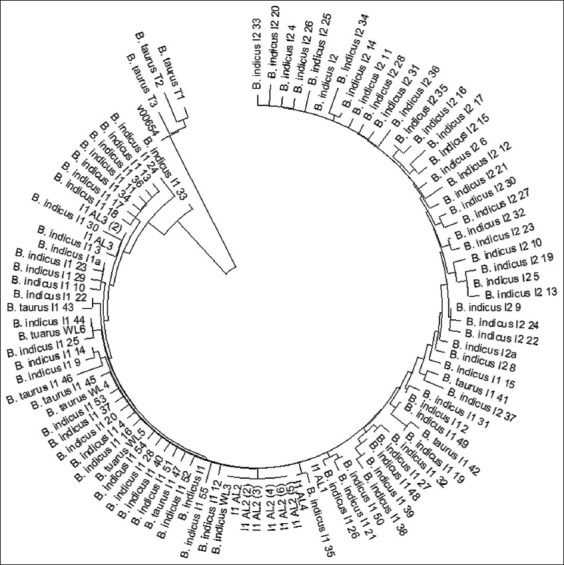
Phylogenetic tree with Neighbor-Joining method (1000× bootstrap) of D-loop mitochondrial DNA between four haplotypes (I1, I1_AL2, I1_AL3, and I1_AL4) of A Luoi Yellow cattle population and reference haplotype sequences of *Bos indicus* and *Bos taurus* cattle.

In the present study, we determined mtDNA haplotypes in 46 animals of the A Luoi Yellow cattle by sequence analysis of a 240-bp segment of the hypervariable D-loop region of bovine mtDNA from position 16049 to 16248 according to the sequence of V00654 (*Bos taurus*). The results indicated that the A Luoi Yellow cattle possess four haplotypes that belong to the indicine-type haplogroup of bovine mtDNA (Table-4). The bovine mtDNA haplotypes have been clustered into the taurine (*B. taurus*) type, which includes the T1’2’3’, T1, T2, T3, T4, and T5 haplogroups, and the indicine (*Bos indicus*) type, which includes the I1 and I2 haplogroup [34]. Thirty-seven of 46 A Luoi Yellow cattle possess a single I1 haplotype. Six animals had the same haplotype with one nucleotide substitution from the I1 haplogroup, as previously reported by Jia *et al*. [35] for the Chinese cattle breed. The remaining three animals possess two new haplotypes not yet observed in other breeds of cattle. The nucleotide sequences of these new haplotypes showed only one nucleotide substitution from the I1 haplotype; thus, these haplotypes were classified into the I1 haplogroup (Table-4 and Figure-1). We tentatively designated these new haplotypes I1_AL3 and I1_AL4.

**Table-4:** Haplotypes of the mtDNA D-loop region observed in A Luoi Yellow cattle.

IDs/haplotypes		Base position																												
		1	1	1	1	1	1	1	1	1	1	1	1	1	1	1	1	1	1	1	1	1	1	1	1	1	1	1	1	1
		6	6	6	6	6	6	6	6	6	6	6	6	6	6	6	6	6	6	6	6	6	6	6	6	6	6	6	6	6
		0	0	0	0	0	0	0	0	1	1	1	1	1	0	1	1	1	1	1	1	1	1	2	2	2	2	2	2	2
		4	5	5	5	7	7	8	8	0	0	1	1	1	1	2	2	3	3	3	4	4	9	0	0	2	3	3	4	4
		9	0	7	8	4	7	2	4	2	9	3	6	7	9	1	2	0	7	8	3	7	6	0	3	9	0	2	7	8
L277331^1^	I1	T	C	A	T	C	T	A	T	A	C	C	C	A	C	A	C	C	C	C	-	C	A	A	A	G	A	C	T	T
V00654^2^	T3	C	.	G	C	T	.	G	C	G	T	T	T	G	T	G	T	T	T	T	A	T	G	-	.	A	.	T	C	C
AL (*n*=37)	I1	.	.	.	.	.	.	.	.	.	.	.	.	.	.	.	.	.	.	.	.	.	.	.	.	.	.	.	.	.
AL (*n*=6)	I1-AL2	.		.	C	.	.	.	.	.	.	.	.	.	.	.	.	.	.	.	.	.	.	.	.	.	.	.	.	.
AL (*n*=2)	^3^I1-AL3	.	T	.		.	.	.	.	.	.	.	.	.	.	.	.	.	.	.	.	.	.	.	.	.	.	.	.	.
AL (*n*=1)	^3^I1-AL4	C	.	.	.	.	.	.	.	.	.	.	.	.	.	.	.	.	.	.	.	.	.	.	.	.	.	.	.	.

Reference sequences of

2 *Bos taurus* and

1 *Bos indicus*,

3 New haplotype; AL=A Luoi Yellow cattle, mtDNA=mitochondrial DNA

**Figure-2:**
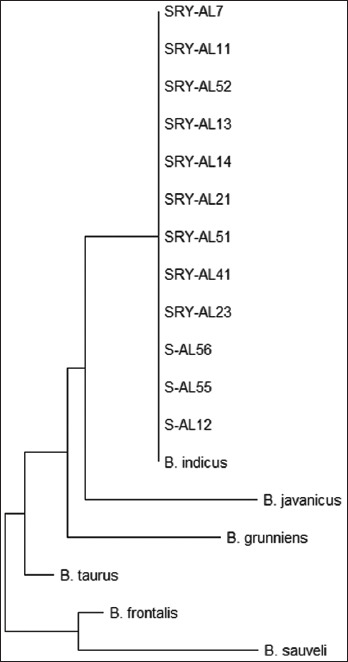
Phylogenetic tree of Y chromosome-specific gene in A Luoi Yellow cattle and other cattle breeds (*Bos taurus*, *Bos indicus*, *Bos frontalis*, *Bos grunniens*, *Bos sauveli*, and *Bos javanicus*).

We also determined the Y-chromosome haplotype of the A Luoi Yellow cattle by sequencing the 1062-bp segment of SRY on the Y chromosome. The Y-chromosome haplotype of cattle can be classified into *B. taurus*, *B. indicus*, *Bos frontalis*, *Bos grunniens*, *Bos sauveli*, and *Bos javanicus* by nine SNPs in this segment [23]. As shown in Table-5 and Figure-2, the A Luoi Yellow cattle have a zebu type Y-chromosome haplotype.

**Table-5:** Haplotypes of the *SRY* gene on the Y chromosome of the A Luoi yellow cattle.

Haplotypes	Position								
	1	1	1	1	1	2	2	2	2
	7	8	9	9	9	0	1	1	2
	4	8	2	4	7	6	0	4	2
	8	4	2	5	2	0	1	5	5
DQ336526 (*Bos taurus*)	G	A	A	C	-	A	C	T	T
DQ336527 (*Bos indicus*)	T	.	.	.	.	.	T	.	.
DQ336528 (*Bos javanicus*)	T	.	.	.	.	G	.	C	.
DQ336531 (*Bos grunniens*)	T	C	.	G	.	.	.	.	.
DQ336530 (*Bos frontalis*)	T	.	G	.	.	.	.	.	T
EF693888 (*Bos sauveli*)	T	.	.	.	T	.	.	.	T
A Luoi Yellow cattle (*n*=12)	T	.	.	.	.	.	T	.	.

SRY=Y chromosome-specific gene

These findings indicate that the A Luoi Yellow cattle originated from indicine cattle from both paternal and maternal lines.

## Discussion

A Luoi Yellow cattle has a small body size; hence, during the past decades, breeding programs have focused on improving this trait. Growth traits are complex and are controlled by multiple genes. It is therefore important for the application of marker-assisted selection to increase body size and carcass weight in the population. *PLAG1* is one of the main genes that play a significant role in the regulation of growth traits [8, 36]. Numerous studies have demonstrated that *PLAG1* gene is significantly associated with growth traits in domestic animals. For example, *PLAG1* gene expression has a certain effect on the length of limb bones in pigs [37]. In sheep, Indel mutations of the *PLAG1* gene were significantly associated with body weight and morphometric traits [38, 39]. In goats, the 15-bp insertion/deletion (Indel) mutation of *PLAG1* is associated with the regulation of important growth characteristics [40]. In cattle, recent studies have found that mutations, including SNPs, Indels, and copy number variant (CNV) in *PLAG1* are associated with growth traits [27, 41, 42]. The polymorphism (*PLAG1* Del_19bp) detected in the intron of chromosome 14 was related to the body size of cattle, but its frequency distribution was quite different. According to Zhou *et al*. [27] the D allele increases the height of cattle, and the prevalence of the D allele is correlated with latitude. In this study, the frequency of D alleles in the A Luoi Yellow population was low, which may be due to the small number of observed individuals, the high altitude, and the low latitude of the A Luoi district.

In addition to *PLAG1*, *SIRT1* and *SIRT2* belong to class I sirtuin genes, which play crucial roles in regulating lipid metabolism, cellular growth, and metabolism, suggesting that they are potential candidate genes affecting body measurement traits in humans and animals [13, 43–45]. In humans, *SIRT1* rs2273773, rs3740051, and rs3758391 are associated with weight, height, body fat, and albumin levels [43]. In sheep, two SNPs (g.3148 C > T and g.3570 G > A) in *SIRT1* and one SNP (g.8074 T > A) in *SIRT2* significantly influenced growth-related traits [44]. In cattle, the SNPs g.17333C > T and g.17578A > G of *SIRT2* have significant effects on the 18-month-old body weight of the Nanyang cattle population [44]. A novel 7-bp indel of *SIRT2* is involved in body length in Chinese Jiaxian cattle [13]. *SIRT1 g*.−382G > A was significantly associated with dressing percentage, meat percentage, and triploid and ribeye weight in Luxi cattle [28].

In our study, although there was only one homozygous cattle carrying the CC-interesting genotype of *SIRT2* detected in the population and even no homozygous animal carrying AA genotype of *SIRT1*, the remaining few heterozygous individuals are useful for future breeding to increase body size in A Luoi Yellow cattle.

The increasing demand for beef consumption requires attention not only to quantity but also to quality. The improvement of these traits through marker-assisted selection has been well-documented during the past decades [4, 46, 47]. Tenderness is one of the main characteristics of meat because it determines price and consumers’ willingness to buy [48, 49]. In particular, genetic factors were found to control 54% of meat tenderness [12]. In the case of the *CAST* and *CAPN1* genes, the influence on the variability of meat tenderness is >40%, and there were at least 175 English language papers from 1993 to 2021 related to the effect of *CAPN1* and *CAST* with/without their SNPs in cattle [49]. *CAST* and *CAPN1* participate in the proteolysis of myofibrillar proteins during the post-mortem storage of carcasses and cut meat at refrigerated temperatures [12]. Polymorphism of *CAST* g. 2959 A>G revealed a strong association with shear force at 7-day postmortem in the taurine, taurine x indicine, and indicine populations [11, 25]. Similarly, a significant association between *CAPN1* g. 4684 C>T and shear force has been observed in various cattle breeds. AA and TT animals had lower shear forces in the taurine and indicine populations [26]. In our study, the frequency of AA genotypes of *CAST* g. 2959 A>G in the A Luoi Yellow cattle population accounted for 20%. However, only heterozygous *CAPN1 g*. 4684 C > T animals were detected in this population. Moreover, *LEP* is a cytokine-like hormone proven to be involved in diverse biological processes. In livestock, *LEP* plays a key role in regulating and controlling the productive performance of animals, including feed intake, body weight homeostasis, energy balance, and fertility [31, 32, 50]. Numerous SNPs, such as *LEP* 580, *LEP* 963, *LEP* 357, *LEP* 1759, *LEP* Y7F, *LEP* A80V, and *LEP* R25C, were detected to be associated with milk fat, body weight, carcass quality, reproduction, and immune responses [30, 32, 51, 52]. *LEP* R25C, a non-synonymous mutation that changes amino acids from arginine to cysteine, is associated with several important traits such as body composition, lactation, reproduction, immunity, fatty acid composition, carcass quality, and milk quality. Giblin *et al*. [53] suggested that *LEP* R25C was significantly associated (p < 0.05) with milk fat and protein concentrations, calving difficulty, and gestational length. Kawaguchi *et al*. [50] showed that *LEP* R25C had a significant effect on the C18:0 and C14:1 ratio in Japanese black cattle. Kononoff *et al*. [31] showed that compared with cattle of the CC genotype, those of the TT genotype had higher 12^th^-rib fat, calculated yield grade, and hot carcass weight gain. Fathoni *et al*. [33] reported that in Kebumen Ongole-grade cattle, animals with the CT genotype had a higher weaning chest circumference than those with the CC genotype. Our present study revealed that the genotype frequencies of CT and TT in the A Luoi Yellow cattle were slightly high at 0.39 and 0.15, respectively. These findings provide insights into the genetic characteristics of meat tenderness and growth traits of A Luoi Yellow cattle in Vietnam.

Furthermore, genetic variation provides essential information for the management of gene resources and mating orientation. Nguyen *et al*. [54] reported that Yellow cattle in An Giang, a southern province of Vietnam, have genetic diversity from maternal lineage and possess the indicine I2 haplogroup of mtDNA, which has origins in Northern Asia and is rarely found in South-east Asian countries. Le *et al*. [55] reported no haplotypes in haplogroup I2 in the A Luoi Yellow cattle. Thus, we performed more experiments to determine whether this cattle population has the I2 haplotype. However, in this study, we only identified other haplotypes of I1 that were not previously observed. In addition, A Luoi Yellow cattle are raised by natural grazing and mating. Hence, Y-chromosome markers are particularly relevant for investigating male introgressions in A Luoi Yellow cattle that live in herds. Verkaar *et al*. [56] reported that crossing wild bovines into domestic populations may create animals with unique properties. For example, Chinese yakow is a yak–taurine hybrid, whereas the South-east Asian Selembu dairy and beef cattle result from *B. frontalis–B. indicus* crossings. Le *et al*. [19] previously indicated a gene flow of taurine cattle from the paternal line to the Yellow cattle population in Quang Tri province, Vietnam. Therefore, in this study, we investigated male introgression in A Luoi Yellow cattle by comparing the Y chromosomal haplotypes of different bovine breeds, including *B. taurus*, *B. indicus*, *B. frontalis*, *B. grunniens*, *B. sauveli*, and *B. javanicus*, with those of A Luoi Yellow males. The results revealed that the A Luoi Yellow cattle only possess the zebu type Y haplotype. Together with the mtDNA results, these findings indicate that the A Luoi Yellow cattle have low genetic diversity from the paternal and maternal lineages, suggesting consideration in conservation and mating orientation.

## Conclusion

We clarified the paternal and maternal origins of A Luoi Yellow cattle and determined the presence of favorable alleles associated with increased meat tenderness and body size. This information will be useful for future breeding programs of A Luoi Yellow cattle and for the positive development of this beef brand.

## Data Availability

The supplementary data, including PCR-RFLP images, can be available from the corresponding author upon a reasonable request.

## Authors’ Contributions

TNAL: Designed the study, performed sampling and DNA extraction, sequenced the mtDNA, analyzed data from PCR-RFLP and sequencing, and drafted the manuscript. HTD: Performed sampling and DNA extraction and carried out PCR-RFLP of the *CAST* and *CAPN1* genes. PTLL: Conducted sampling and DNA extraction and carried out PCR-RFLP of the *LEP* and *PLAG1* genes. TTN: Performed sampling and DNA extraction and sequenced the *SRY* gene. TTDP: Carried out PCR-RFLP of *SIRT1* and *SIRT2*. PDL and VHN: Designed the study and critically reviewed and revised the manuscript. All authors have read, reviewed, and approved the final manuscript.
